# Muscle recruitment during gait in individuals with unilateral transfemoral amputation due to trauma compared to able-bodied controls

**DOI:** 10.3389/fbioe.2024.1429574

**Published:** 2024-09-23

**Authors:** Alice M. Benton, Diana Toderita, Natalie L. Egginton, Sirui Liu, Pouya Amiri, Kate Sherman, Alexander N. Bennett, Anthony M. J. Bull

**Affiliations:** ^1^ Department of Bioengineering, Imperial College London, London, United Kingdom; ^2^ Academic Department of Military Rehabilitation, Defence Medical Rehabilitation Centre, Loughborough, United Kingdom; ^3^ School of Kinesiology and Health Studies, Faculty of Arts and Science, Queen’s University, Kingston, ON, Canada; ^4^ Dorset Orthopaedic GB, Ringwood, United Kingdom

**Keywords:** gait, unilateral transfemoral amputation, muscle recruitment, musculoskeletal modelling, walking

## Abstract

Individuals with transfemoral lower limb amputations walk with adapted gait. These kinetic and kinematic compensatory strategies will manifest as differences in muscle recruitment patterns. It is important to characterize these differences to understand the reduced endurance, reduced functionality, and progression of co-morbidities in this population. This study aims to characterize muscle recruitment during gait of highly functional individuals with traumatic transfemoral amputations donning state-of-the-art prosthetics compared to able-bodied controls. Inverse dynamic and static optimisation methods of musculoskeletal modelling were used to quantify muscle forces of the residual and intact limb over a gait cycle for 11 individuals with traumatic transfemoral amputation and for 11 able-bodied controls. Estimates of peak muscle activation and impulse were calculated to assess contraction intensity and energy expenditure. The generalized estimation equation method was used to compare the maximum values of force, peak activation, and impulse of the major muscles. The force exhibited by the residual limb’s iliacus, psoas major, adductor longus, tensor fasciae latae and pectineus is significantly higher than the forces in these muscles of the intact contralateral limb group and the able-bodied control group (*p* < 0.001). These muscles appear to be recruited for their flexor moment arm, indicative of the increased demand due to the loss of the plantar flexors. The major hip extensors are recruited to a lesser degree in the residual limb group compared to the intact limb group (*p* < 0.001). The plantar flexors of the intact limb appear to compensate for the amputated limb with significantly higher forces compared to the able-bodied controls (*p* = 0.01). Significant differences found in impulse and peak activation consisted of higher values for the limbs (residual and/or intact) of individuals with transfemoral lower limb amputations compared to the able-bodied controls, demonstrating an elevated cost of gait. This study highlights asymmetry in hip muscle recruitment between the residual and the intact limb of individuals with transfemoral lower limb amputations. Overall elevated impulse and peak activation in the limbs of individuals with transfemoral amputation, compared to able-bodied controls, may manifest in the reduced walking endurance of this population. This demand should be minimised in rehabilitation protocols.

## Introduction

In 2017 there was an estimated 28.9 million people living globally with unilateral lower limb amputations due to traumatic causes (McDonald et al., 2021). The physiological demand of gait is greater in this population, which leads to reduced endurance as evidenced by a 29% reduction in the 6-min walking test (Linberg et al., 2013). Ex-military individuals with transfemoral amputation are likely to be the gold standard of functional ability, as they are young, experience comprehensive rehabilitation, have high levels of fitness preinjury and are fitted with state-of-the-art prosthetics (Jarvis et al., 2021). However, even with these positive factors, the oxygen cost of gait at self-selected speeds is 20% higher for ex-military personnel with unilateral transfemoral amputations (n = 10, average age = 29 years) compared to able-bodied controls (n = 10, average age = 30 years) (Jarvis et al., 2017). These findings are supported by another study which controlled for walking speed and found an 24.1%–24.2% increase in oxygen consumption for young, fit, individuals with traumatic unilateral amputations donning micro-processor knees (n = 8, six male, two female, average age = 22.5 years) compared to an able-bodied control group (Chin et al., 2003). This elevated oxygen cost has been found to be even more extreme, 55%, when observing a population of older civilian individuals with transfemoral amputation (n = 60, average age = 51.1 years) donning mechanical knees compared to their age-matched able-bodied control group (n = 10, average age = 51 years) (Carse et al., 2020). Oxygen cost during gait in individuals with lower limb amputation has been found to be higher in transfemoral compared to trans-tibial amputation (Göktepe et al., 2010).

Co-morbidities such as lower back pain and osteoarthritis are prevalent in individuals with unilateral transfemoral limb amputation (Silverman et al., 2023): 47.7%–76.6% of individuals with unilateral amputations experience lower back pain, compared to 1%–37% in an able-bodied population (Sivapuratharasu et al., 2019). These co- morbidities are detrimental to quality of life (Silverman et al., 2023).

Unilateral transfemoral limb loss, muscle loss, and loss of control mechanisms result in compensatory strategies in gait biomechanics, including kinematics, kinetics (Seroussi et al., 1996; Jarvis et al., 2021; Silverman et al., 2023), and muscle activation timing (Mehryar et al., 2021; Wentink et al., 2013) which may lead to muscular loading conditions associated with loss of muscular endurance (Fang et al., 2007), functional deficit (Slater et al., 2022) and the risk of developing secondary conditions (Ding et al., 2021). Though it is evident that there is a need to improve these rehabilitation outcomes, muscle recruitment in the residual limb and intact limb, compared to an able-bodied control group has not been quantified in this young, fit, military cohort. Musculoskeletal modelling is a non-invasive method of estimating muscle and joint contact forces from motion data, providing insight into pathological and non-pathological gait. Within research of biomechanics of individuals with lower limb amputation, musculoskeletal modelling has been used for several applications, such as, to assess prosthetic device function (Pickle et al., 2017), understand the progression of musculoskeletal pathologies (Ding et al., 2021) and evaluate surgical technique (Ranz et al., 2017). One study utilised musculoskeletal modelling to quantify muscle forces during walking to identify the contribution to centre of mass acceleration for the intact and residual limb of individuals with unilateral transfemoral amputations and found significant differences in force magnitude between the limbs, as well as compensatory mechanism adopted by the muscles in the intact limb to accelerate the centre of mass. However, this study was looking at a small sample size (n = 6) donning a mix of microprocessor and non-microprocessor knees, and did not include a control group in their analysis. Additionally, it was also solely looking at the stance phase of gait (differences in muscle recruitment has been found during the swing phase in transtibial amputees (Ding et al., 2023)) and scaled all subjects from a generic musculoskeletal model (Harandi et al., 2020).

The objective of this study was to quantify muscle recruitment, with force magnitude and metrics associated with endurance, during the whole gait cycle in the intact and residual limb of individuals with traumatic unilateral transfemoral (UTF) amputation who have received high quality intensive rehabilitation and advanced prosthetics, and compare this to able-body equivalents, using validated musculoskeletal modelling methods (Toderita et al., 2021b; Ding et al., 2023). This investigation will contextualise muscle recruitment into known kinematic and kinetic adaptations of this population (Jarvis et al., 2021) to inform future rehabilitation research aimed to improve walking endurance, functional ability, alleviate the risk of co-morbidities and therefore improve quality of life.

## Methods

### Participant demographics

This study focused on 11 male young/middle-aged individuals with unilateral transfemoral (UTF) amputation due to traumatic injuries donning state-of -the-art prosthetics following intensive rehabilitation. 9 of the 11 participants were ex-military personnel. This demographic can be taken as a model of high-functionality in persons with UTF limb loss (Jarvis et al., 2021). This study considered male participants only due to the lack of availability of anatomical datasets for female persons with UTF amputation. 11 able-bodied (AB) participants were selected as a control group, matched to the UTF group for gender, age and height (Table 1). The able-bodied dataset has been previously published (Long et al., 2017; Ding et al., 2019). The able-bodied group were significantly lighter (body mass) than individuals with UTF amputations, which is consistent with the literature in this predominantly military group (McMenemy et al., 2023). Ethical approval was received from the institutional ethics review board and written informed consent obtained from the participants.

**TABLE 1:** Study participant demographics.

Group	Participant code	Sex	Age (years)	Body mass with prostheses (kg)	Calculated intact mass (kg)*	Height (m)	Level	Prosthetic knee
UTF (n = 11)	1	M	47	93.8	95.4	1.76	UTF	Ottobock Genium X3
	2	M	57	91.8	97.8	1.76	UTF	Ottobock C-leg
	3	M	58	64.4	65.5	1.64	UTF	Endolite Orion
	4	M	52	91	94.2	1.78	UTF	Ottobock C-leg
	5	M	32	88.7	94.6	1.74	UTF	Ottobock Genium X3
	6	M	49	82.7	86.4	1.77	UTF	Ottobock Genium X3
	7	M	47	94.1	95.5	1.77	UTF	Ottobock Genium
	8	M	31	74.3	77.3	1.75	UTF	Ottobock Genium X3
	9	M	36	109.3	114.3	1.89	UTF	Ottobock Genium X3
	10	M	39	133.3	139.4	1.90	UTF	Blatchford KX06
	11	M	36	91.1	96.3	1.83	UTF	Ottobock Genium X3
	Mean	—	44.0	92.2	96.0	1.78	—	—
	SD	—	9.7	17.9	18.9	0.07	—	—
AB (n = 11)		M						
	Mean	—	42.2	79.5		1.79	—	—
	SD	—	11.0	93		0.07	—	—
	P-value	—	0.74	0.04**	0.02**	0.85	—	—

*calculated using Equation 5; **t-test between UTF participants and AB participants.

### Experimental data

Reflective markers were attached to anatomical landmarks of the pelvis, the left and right lower limbs as well as marker clusters on the thighs and shanks (Table 2). Three gait trials at a self-selected walking speed and one static trial were collected for each participant in a motion capture system. For UTF participants 1 to 4 this motion capture system consisted of ten cameras (VICON, Oxford Metrics Group, United Kingdom) and two force plates (Kistler Type 9286B, Kistler Instrumented AG, Winterthur, Switzerland). The marker 3D displacement and ground reaction forces were sampled at 120 and 960 Hz, respectively. For participants 5 to 11 this consisted of twenty cameras (VICON, Oxford Metrics Group, United Kingdom) and six Optima force plates (AMTI Force and Motion, Massachusetts, United States). The marker 3D displacement and ground reaction forces were sampled at 100 and 1,000 Hz, respectively. For the previously published able-bodied trials this consisted of ten cameras (VICON, Oxford Metrics Group, United Kingdom) and two force plates (Kistler Type 9286B, Kistler Instrumented AG, Winterthur, Switzerland), sampled at 100 Hz and 1,000 Hz respectively (Long et al., 2017).

**TABLE 2:** Optical motion marker labels and locations.

Marker label	Anatomical location
FCC	Calcaneus
FMT	Tuberosity of the fifth metatarsal
FM2	Head of the second metatarsal
TF	Additional marker placed on foot
FAM	Apex of the lateral malleolus
TAM	Apex of the medial malleolus
C1, C2, C3 (clusters)	Additional markers placed on the shank segment
FLE	Lateral femoral epicondyle
FME	Medial femoral epicondyle
T1, T2, T3 (clusters)	Additional markers placed on the thigh segment
RASIS	Right anterior superior iliac spine
LASIS	Left anterior superior iliac spine
RPSIS	Right posterior superior iliac spine
LPSIS	Left posterior superior iliac spine

## Musculoskeletal modelling

### General modelling method

The FreeBody musculoskeletal model (Cleather and Bull, 2015) was utilised to quantify the muscle forces for the participants with UTF amputation and the able-bodied group. FreeBody 2.1 has been validated against in-vivo knee contact forces for able-bodied individuals (Ding et al., 2016) and for muscle activations against EMG for individuals with bilateral transfemoral amputation (Toderita et al., 2021b) and unilateral transtibial amputation (Ding et al., 2023). FreeBody 2.1 is a segment based musculoskeletal model with four rigid bodies including the foot, shank, thigh and pelvis. FreeBody 2.1 uses quaternion algebra to perform inverse kinematics to calculate joint kinematics, inverse dynamics with wrench formulations to estimate net forces and moments, and then a one-step static optimisation to predict muscle forces and joint loading for the captured gait trial. The static optimisation minimises the sum of cubed muscle activations following the objective function Equation 1 (Crowninshield and Brand, 1981), where J is the sum of the cubed muscle activations, $F_{i}$ is the instantaneous force of muscle element i, $F_{\max}^{i}$ is the maximum force potential of the muscle element i, and n is the number of muscle elements of the model. Ligament force contribution is assumed insignificant to net moments.

$$J=\sum_{i=1}^{n}{\left(\frac{F_{i}}{F_{\max}^{i}}\right)}^{3}$$ (1)

The maximum force potential of each muscle element is calculated following Equation 2 which is a multiplication of the physiological cross-sectional area (PCSA) of the muscle element i and the assumed maximum muscle stress ( σ ) which is 31.39 N $/{cm}^{2}$ (Yamaguchi, 2005).

$$F_{\max}^{i}={PCSA}_{i}\;\times \;\sigma$$ (2)

The PCSA was calculated following Equation 3 where muscle volume ( $\left.V_{m}\right)$ was acquired from rendered 3D geometry of the muscles (details described below), fibre length to muscle length ratio ( $\frac{L_{f}}{L_{m}}$ ), pennation angle ( θ ) and optimal sarcomere length ( $L_{s}$ ) were taken from the literature. In the cases where there were no values found in the literature, θ was set to 0, $\frac{L_{f}}{L_{m}}$ to 1 and $L_{s}$ to 2.7 μ m (Lieber et al., 1994; Ding et al., 2019).

$$PCSA=\frac{V_{m}\times \cos\theta }{L_{m}\times \frac{L_{f}}{L_{m}}\times \frac{2.7}{L_{s}}}$$ (3)

### Able-bodied limb and intact limb model

FreeBody is a unilateral model, so the individual limbs of a participant are modelled separately. The limbs of the able-bodied participants and the intact limbs of the participants with transfemoral amputations are modelled in an identical manner. This model consists of 163 muscle elements representing 38 muscles. The muscles are modelled as an ideal force generator, where the force is proportional to the maximum force potential. The anatomical measures (muscle and joint geometries) required for the static optimisation, as previously described, are acquired from a previously published anatomical able-bodied atlas of anatomical datasets (Ding et al., 2019). Each dataset contains origin, insertion and via points of the 163 muscle elements. The dataset defines the centres of joint rotation, bone geometries, and the wrapping surfaces of the muscles with curved lines of actions. This information was originally digitised from MRI scans of able-bodied participants, following the methodology of (Horsman et al., 2007). The method of Ding et al. (2019), was used to select an appropriate anatomical dataset from the atlas and scale for each participant’s dimensions following the regression model in Equation 4 to minimize error in hip contact forces, where RMSD represents the root mean square difference, ΔLL rthe difference in limb length, ΔMass the difference in body mass and Δgender the difference in gender.

RMSD=9.60+0.38 × ΔLL+0.10 × ΔMass+2.64 × Δgender (4)

The segment parameters (mass, centre of m ass location and moment of inertia) were calculated from De Leva’s method (De Leva, 1996). For the intact limb, the segment parameters were calculated using the adjusted intact weight of the participant if they did not have the transfemoral amputation. The adjusted intact weight was estimated using Equation 5 (Tzamaloukas et al., 1994) where *W*_I_ is the estimated intact mass, $w_{a}$ is the weight without the prosthetic device, $\sum \frac{∆W}{W_{I}}$ is the percentage of bodyweight lost due to amputation.

$$W_{I}=\frac{W_{a}}{1-\sum \frac{∆W}{W_{I}}}$$ (5)

### Residual + prosthetic limb model

The UTF residual limb model consists of 92 muscle elements, representing 21 muscles. The anatomical geometries for the residual limb models were acquired from a previously published atlas of datasets of residual limbs of participants with bilateral transfemoral amputations (Toderita et al., 2021b). The method to acquire these muscle and bone geometries is the same as the intact and able-bodied limbs. Anatomical dataset selection and scaling followed Equation 6 (Toderita et al., 2021b), where RMSD represents the root mean square difference, ΔPW difference in pelvis width, ΔBMI difference in body mass index (from the adjusted body mass, Equation 5), and Δratio residual limb length to pelvis ratio.

RMSD=36.82+6.55 × ΔPW+2.85 × ΔBMI+0.29 × Δratio – 0.38 × ΔBMI × ΔPW (6)

The inertial properties of the residual limb model represent the residual and prosthetic limb configuration. The mass properties of the prosthetic foot, knee and socket had been previously determined (Toderita et al., 2021b), using the reaction board method for the moment of inertia and the moment of equilibrium for the centre of mass location (Smith et al., 2014). For the prosthetic foot and the prosthetic knee, these values are directly inputted into the model for the foot and shank segment respectively. The socket and the residual limb are modelled as one rigid component. The mass of the residual limb was calculated from the estimated mass of the participant’s thigh from the De Leva equations using the adjusted intact mass. This was multiplied by the ratio of residual limb length to calculated intact limb length (according to the adjusted De Leva calculations). The moment of inertia and centre of mass of the residual limb was assumed to be proportional to De Leva and calculated using the residual limb length. The composite thigh segment was the sum of the estimated residual limb mass and the measured mass of the socket and liner. The moment of inertia of composite residual limb and socket segment was calculated using Huygens-Steiner parallel axis theorem. The centre of mass position was taken as an average of the socket and the calculated residual.

## Data analysis and statistical testing

The limbs were grouped into: the residual limb of the participants with UTF group (R), the intact limb of the participants with UTF group (I), and the limb of the able-bodied participants control group (AB).

The hip muscle (iliacus, psoas major, rectus femoris, sartorius, biceps femoris, gluteus maximus, semitendinosus, adductor brevis, adductor magnus, adductor longus, pectineus, gracilis, gluteus medius, gluteus minimus, tensor fascia latae) and plantar flexor muscle (gastrocnemius, soleus) forces of the residual, intact and able-bodied limb groups were compared. For each muscle, the force was calculated by summing the $F^{i}$ of its muscle elements and was normalised to measured body weight plus weight of the prosthetic components (BW). This force was plotted over the gait cycle (initial foot contact to subsequent foot contact); this output will be referred to as ‘force over gait cycle’ throughout this study. The maximum value of force over gait cycle was calculated; this parameter will be referred to as maximum muscle force. Additionally, as muscular endurance is negatively associated with intensity of force production with respect to maximum voluntary contraction (West et al., 1995), activation was estimated by normalising the total $F^{i}$ of the muscle by the maximum force capacity (total $F_{\max}^{i}$ of that muscle) following Equation 2. The maximum value of activation over the gait cycle was calculated; this will be referred to as peak activation throughout this study and is an estimation of contraction intensity. Additionally, force impulse was calculated by finding the area under the ‘force over gait cycle’ curve as an indication of the total muscular energy expended (Bemben et al., 1996). This parameter will be referred to as impulse throughout this study.

A three-way comparison was made of the kinematics and kinetics to assist the interpretation of the muscle recruitment comparison. Kinetics were normalised to BW. The inverse kinematic, inverse dynamic were plotted over the gait cycle. The maximum values of kinematic and kinematic features of gait were calculated. These gait features include maximum anterior pelvic tilt, maximum hip flexion and extension angles, maximum hip abduction and adduction angles, maximum external hip extension and flexion torques, the first and second peak of external hip adduction torque, and the first and second peak of vertical ground reaction force. The self-selected gait speed of the gait cycle was also calculated.

A preliminary student’s t-test was conducted between the left and the right limb of the able-bodied control group. This test showed no significant difference between the two sides in any of the variables (maximum muscle forces, peak activation, impulse and gait features). So, the right and left limb of the able-bodied controls were treated as one group for comparison. For the three-way comparison of maximum muscle force, peak activation, impulse and gait features, generalized estimation equations were used to compare the means of the variables described between the residual limb group, the intact limb group, and the able-bodied limb group (control group), with significance set at 0.05. The generalized estimation equation method accounts for the potential correlation between variables of limbs from the same participant (Wang, 2014). For the two-way comparison of force over gait cycle (between the plantar flexors of the intact and able-bodied limb group), statistical parametric mapping was used with a Friedman with Wilcoxon test with significance set at 0.05 (Pataky et al., 2013). A student’s t-test was conducted between self-selected gait speed of the UTF participants and the able-bodied control group with significance set at 0.05.

## Results

The able-bodied limb group consists of 22 limbs, the residual limb group 11 limbs and the intact limb group 10 limbs due to an error identified in the motion capture data of the intact side of participant 6. These trials have been excluded from the analysis. The residual limbs from this participant were included in the analysis as the generalized estimation equation method accounts for participants and all values were normalised.

## Muscle recruitment during gait

### Maximum muscle force

**FIGURE 1:**
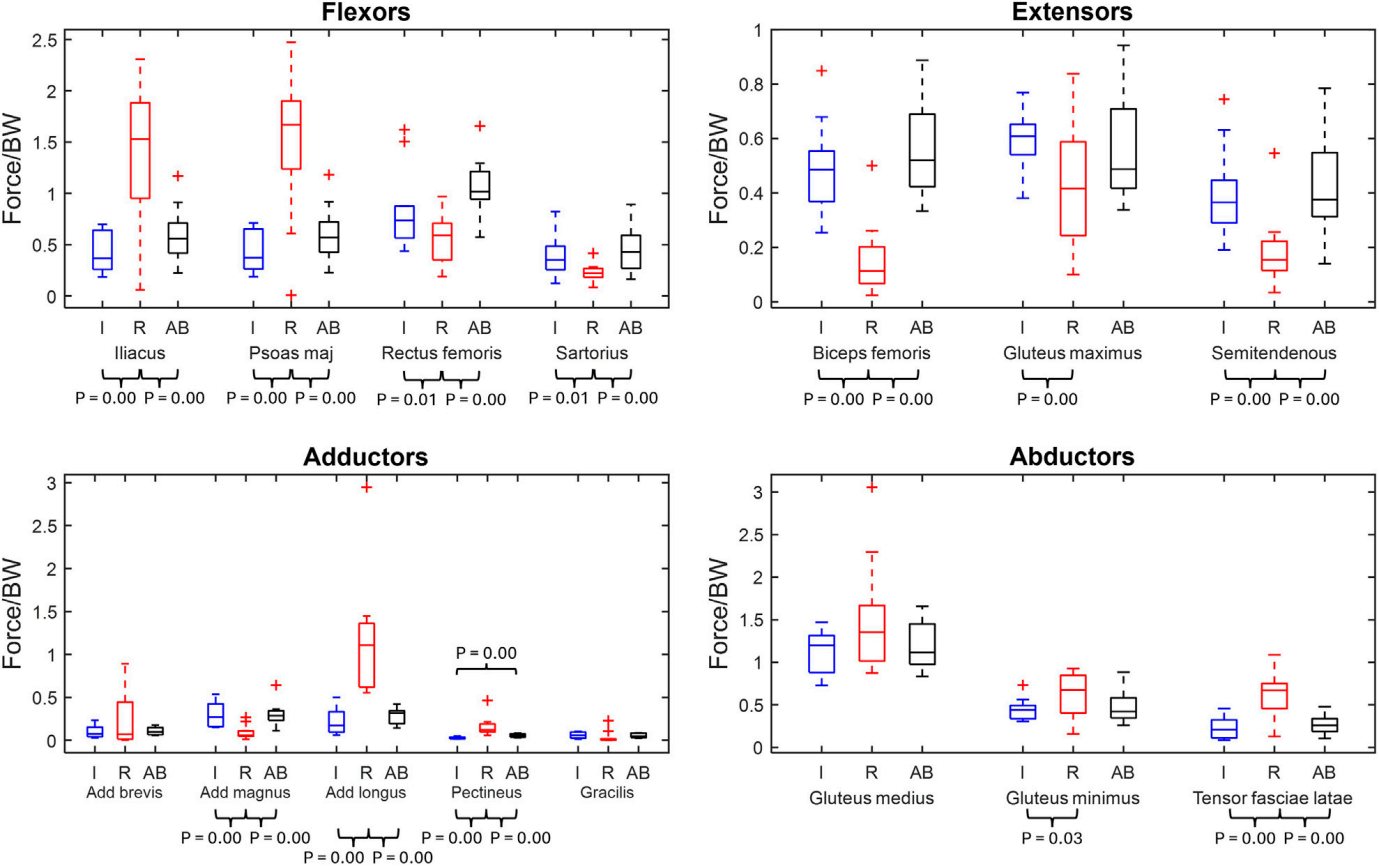
Maximum normalised muscle force box plots of major hip muscles grouped by primary function (min, max, median, 25 and 75 percentiles and outliers). Significant p-values displayed. The muscles are grouped by primary function. Red represents the residual limb group (n = 11) of the individuals with UTF (“R”), blue represents the intact limb group (n = 10) of individuals with UTF (“I”) and black represents the able-bodied limb group (n = 22) (“AB”).

Hip muscles are grouped by primary function and maximum muscle forces are presented in Figure 1, with significant p-values labelled. Figure 1 displays that the largest overall magnitudes produced by the major hip muscles across the groups. The largest values were the residual limb’s gluteus medius, iliacus, and psoas major, which have mean values of 1.49 ± 0.67 N/BW, 1.40 ± 0.72 N/BW, and 1.50 ± 0.72 N/BW, respectively, followed by the able-bodied and intact groups’ gluteus medius at 1.20 ± 0.39 N/BW and 1.14 ± 0.25N/BW.

From the comparison between the residual limb group and the intact limb group, twelve out of fifteen maximum muscle forces were significantly different. Six muscles (iliacus, psoas major, adductor longus, pectineus, gluteus minimus and tensor fasciae latae) were recruited to significantly higher levels in the residual limb group compared to the intact limb group. Six other muscles (rectus femoris, sartorius, biceps femoris, gluteus maximus, semitendinosus, and adductor magnus) exhibited significantly higher maximum muscle force in the intact limb group compared to the residual.

From the residual limb group to able-bodied limb group comparison, ten out of fifteen of the major muscles were recruited to significantly different maximum muscle forces. Five muscles (iliacus, psoas major, adductor longus, pectineus and tensor fasciae latae) had significantly greater maximum muscle force in the residual limb group compared to the able-bodied limb group. Five muscles (adductor magnus, biceps femoris, rectus femoris, sartorius, and semitendinosus) were recruited to higher maximum force in the able-bodied limb group than the residual limb group.

From the intact limb group to able-bodied limb group comparison, only the pectineus exhibited a significantly higher maximum muscle force in the able-bodied limb group, Figure 1.

**FIGURE 2:**
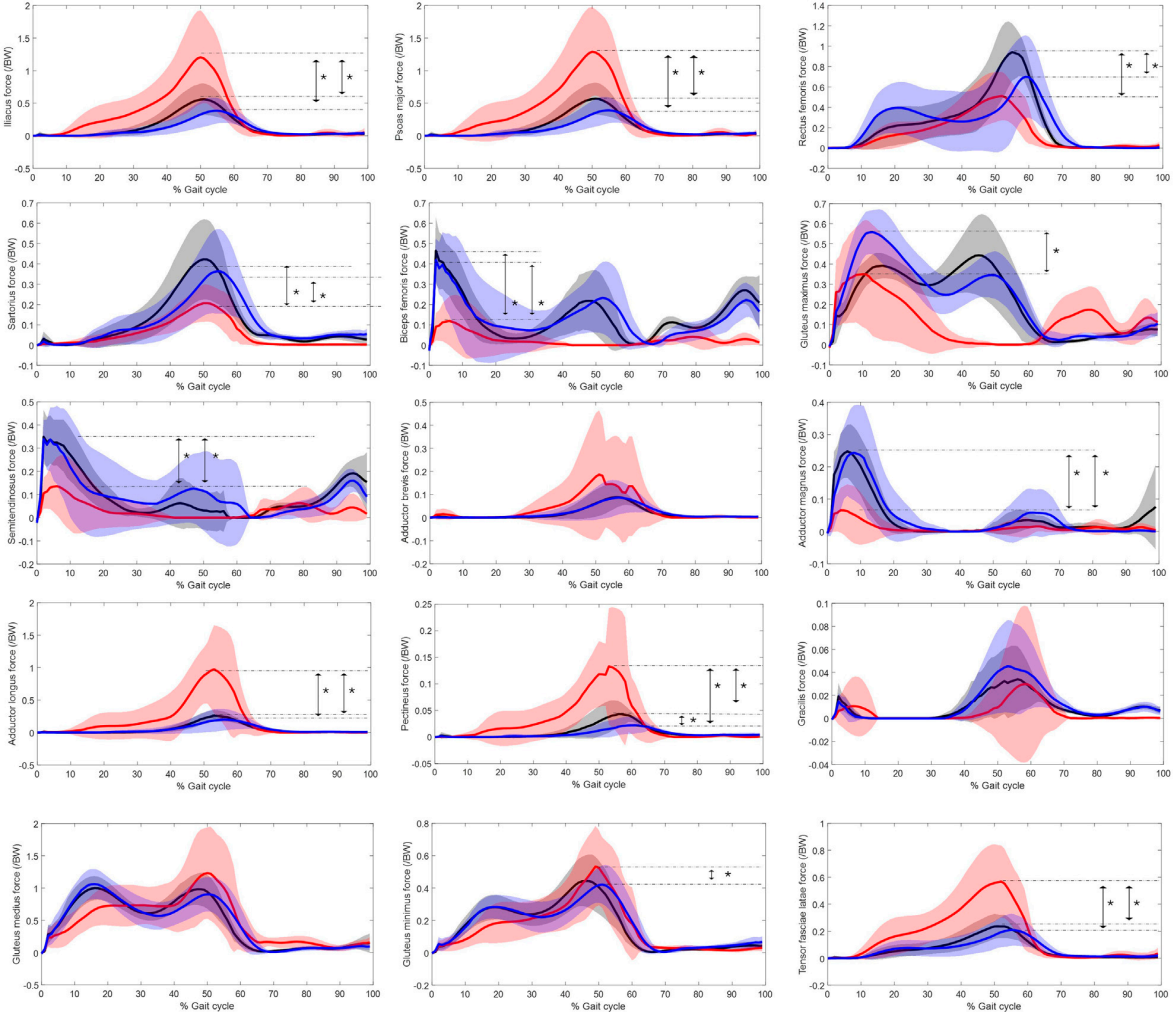
Muscle force normalised to bodyweight of major hip muscles over gait cycle. Red represents the residual limb group (n = 11 ± SD) of the individuals with UTF, blue represents the intact limb group (n = 10 ± SD) of individuals with UTF and black represents the able-bodied limb group (n = 22 ± SD). Arrows and “*” indicate a significant difference in peak value.

The force over gait cycle for the 15 hip muscles are displayed in Figure 2, with the significant differences marked in maximum muscle force labelled.

**FIGURE 3:**
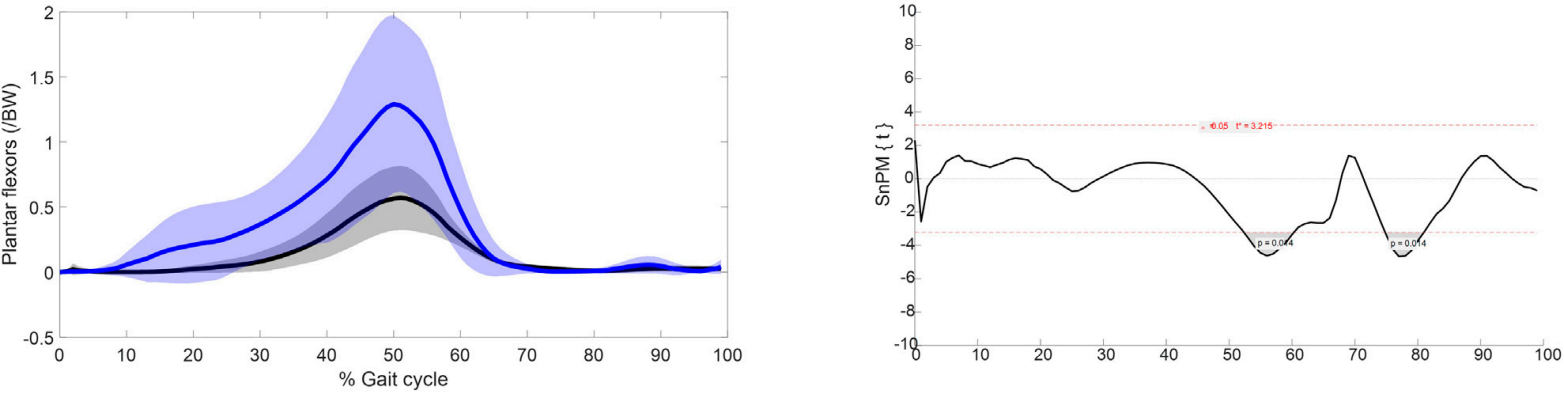
Left: muscle force normalised to bodyweight of the plantar flexors over the gait cycle. Blue represents the intact limb group (n = 10 ± SD) and black represents the able-bodied limb group (n = 22 ± SD). Right: Statistical parametric mapping results.

Statistical parametric mapping identified significant differences in the force over gait cycle of the plantar flexors of the intact limb group and the able-bodied limb group, Figure 3. The plantar flexors were recruited to significantly higher levels in the intact limb group than the able-bodied limb group during 50%–60% and 70%–80% of gait.

### Impulse

**FIGURE 4:**
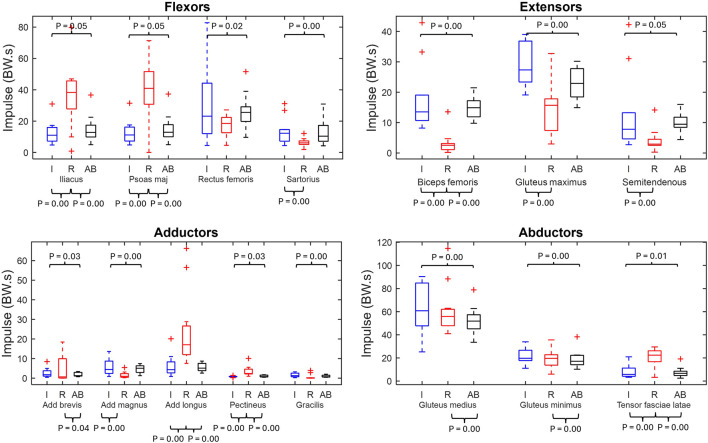
Muscle impulse box plots of major hip muscles grouped by primary function (min, max, median, 25 and 75 percentiles and outliers). Significant p-values displayed. Red represents the residual limb group (n = 11) of the individuals with UTF (“R”), blue represents the intact limb group (n = 10) of individuals with UTF (“I”) and black represents the able-bodied limb group (n = 22) (“AB”).

Figure 4 displays the impulse for the main fifteen hip muscles of the residual limb, intact limb, and able-bodied limb groups.

From the residual limb to intact limb group comparison, ten out of the fifteen of the impulse values were significantly different. Five muscles (iliacus, psoas major, adductor longus, pectineus and tensor fasciae latae) showed significantly higher values of impulse in the residual limb group compared to the intact limb group. While five muscles (sartorius, biceps femoris, gluteus maximus, semitendinosus and adductor magnus) had higher values of impulse in the intact limb group compared to the residual limb group.

From the residual and able-bodied limb group comparison there were nine out of the fifteen significant differences. Eight muscles (iliacus, psoas major, adductor brevis, adductor longus, pectineus, gluteus medius, gluteus minimus and tensor fasciae latae) had a greater impulse in the residual limbs compared to the able-bodied limbs. Whilst the biceps femoris had a greater impulse in the able-bodied limbs than the residual.

From the intact limb group to able-bodied limb group comparison all muscles, apart from the adductor longus, exhibited a significantly greater impulse in the intact limb group than the able-bodied limb group.

### Peak muscle activation

**FIGURE 5:**
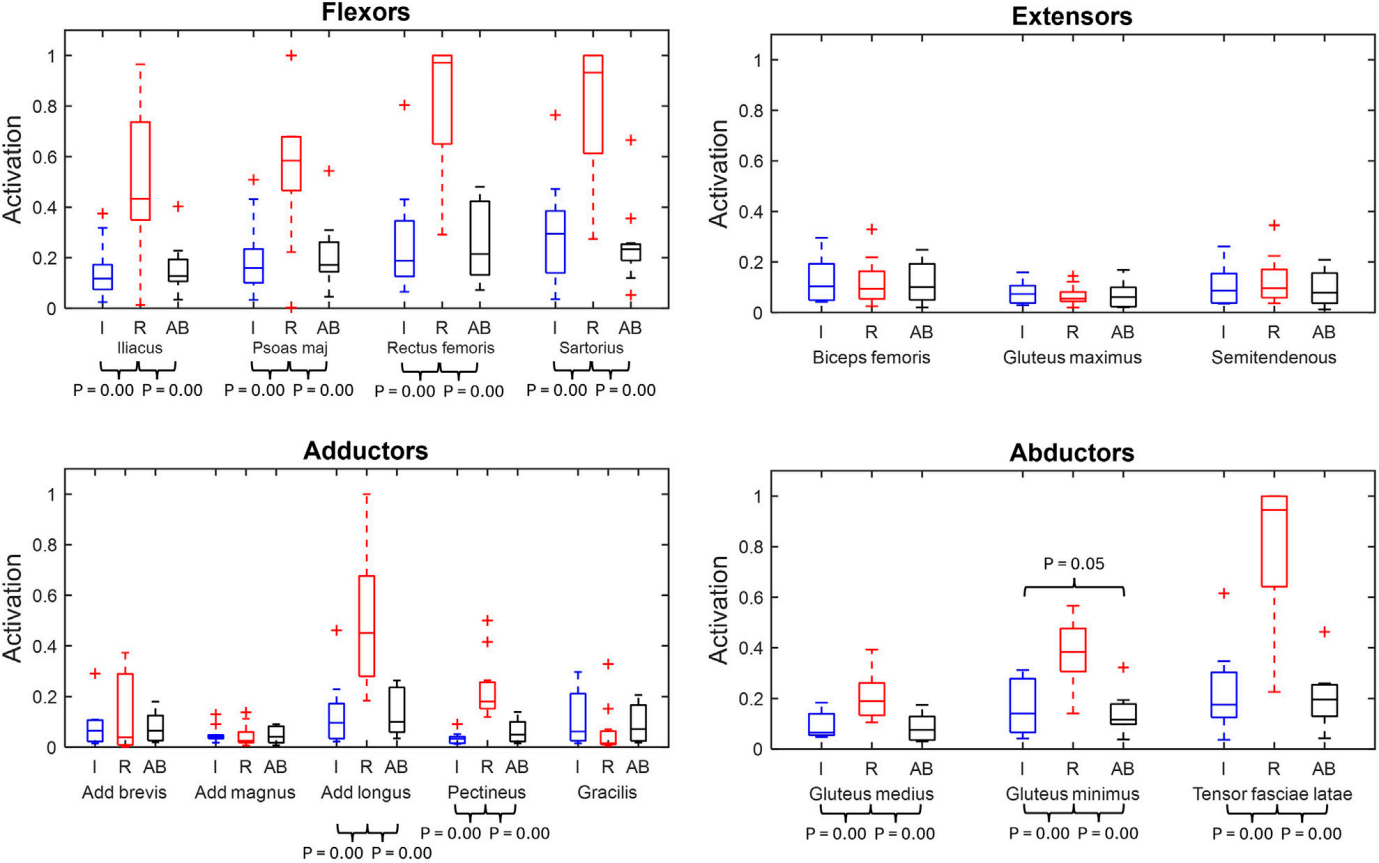
Muscle peak activation box plots of major hip muscles grouped by primary function (min, max, median, 25 and 75 percentiles and outliers). Significant p-values displayed. Red represents the residual limb group (n = 11) of the individuals with UTF (“R”), blue represents the intact limb group (n = 10) of individuals with UTF (“I”) and black represents the able-bodied limb group (n = 22) (“AB’).

Figure 5 displays the peak muscle activations for the main hip muscles for the residual, intact and able-bodied limb group.

From the residual to intact limb group comparison, nine (iliacus, psoas major, rectus femoris, sartorius, adductor longus, pectineus, gluteus medius, gluteus minimus and tensor fasciae latae) of the fifteen muscles were recruited to significantly higher peak activation in the residual limb group compared to the intact.

From the residual limb to able-bodied limb group comparison the same nine (iliacus, psoas major, rectus femoris, sartorius, adductor longus, pectineus, gluteus medius, gluteus minimus and tensor fascia late) of the fifteen muscles were recruited to significantly higher peak activation in the residual than the able-bodied limb group.

From the intact limb group to able bodied limb group comparison the gluteus minimus had significantly greater peak activation in the intact limbs compared to the able-bodied limbs.

## Gait features

**FIGURE 6:**
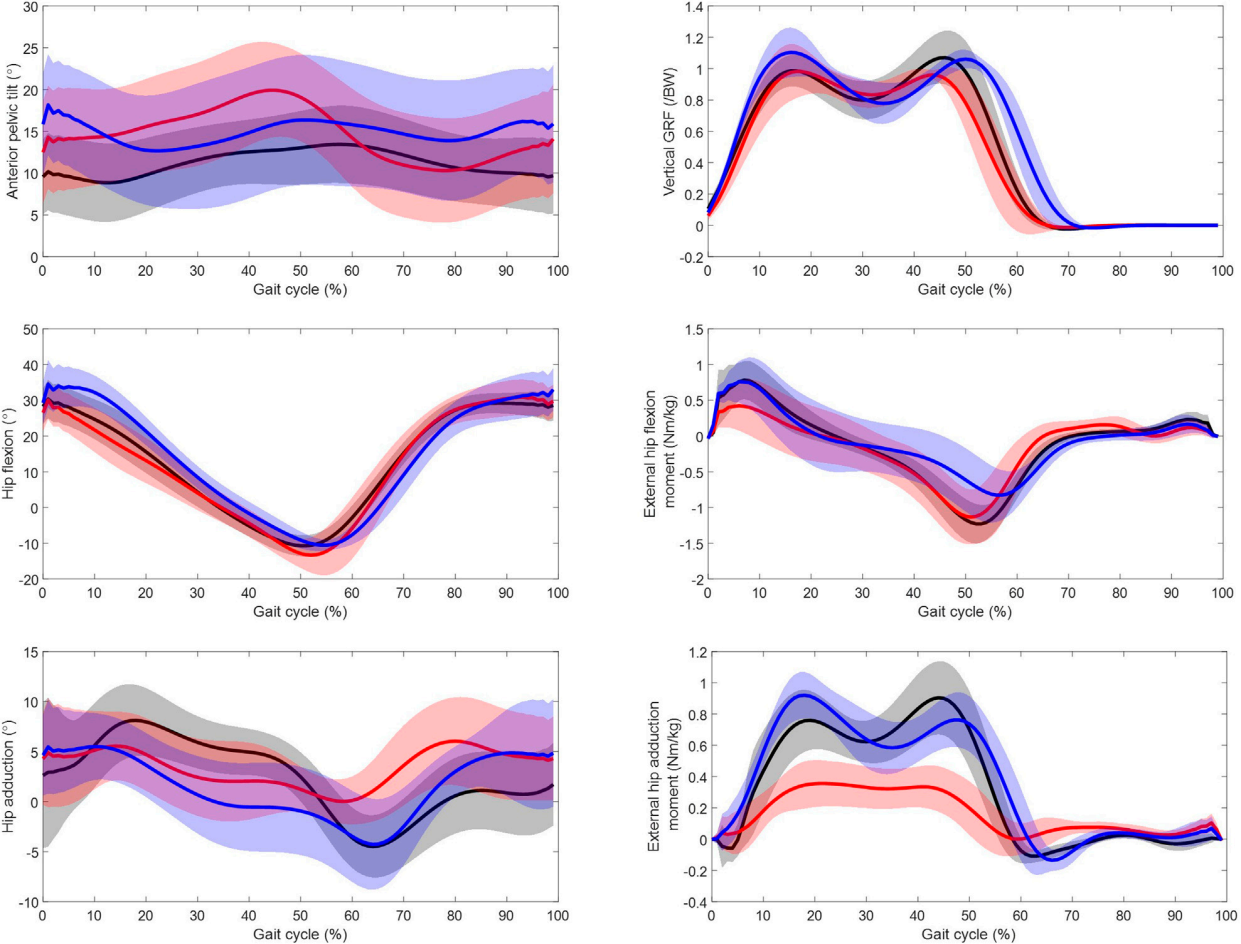
Kinetic and kinematic parameters over gait cycle from initial foot contact (0%–100%) for the residual limb group (red, n = 11 ± SD), the intact limb group (blue, n = 10 ± SD) and the able-bodied control limb group (black, n = 22 ± SD). Solid line represents mean and shaded standard deviation.

Figure 6 displays the anterior pelvic tilt, the vertical ground reaction force, and the sagittal and frontal hip kinematics and moments over the gait cycle for the residual, intact and able-bodied limbs. The angle of maximum anterior pelvis tilt was greater in the residual than the able-bodied (*p* = 0.00). Maximum external hip flexion torque was significantly greater in the intact and able-bodied limbs than the residual limbs (*p* = 0.00 and *p* = 0.04 respectively). The maximum external hip extension torque was lower in the intact limbs compared to the residual and able-bodied limbs (*p* = 0.00 and *p* = 0.04 respectively). For the hip kinematics in the frontal plane, the maximum abduction angle was significantly greater (*p* = 0.00) in the intact and able-bodied limbs compared to the residual limbs. The external hip adduction torque was significantly smaller (*p* = 0.00) in the residual limbs compared to the intact and able-bodied for both peaks. There was no significant difference in self-selected gait speed between the UTF participants and the able-bodied participants which was 1.08 m/s and 1.23 m/s respectively (*p* = 0.20).

## Discussion and conclusion

This is the first study to have quantified normalised muscle force, impulse, and peak activation during gait of individuals with transfemoral amputation of a “high-functioning” demographic due to their comprehensive rehabilitation, high levels of fitness prior to surgery and state-of the-art prosthetics. Comparable self-selected walking speeds of the UTF participants to the matched able-bodied controls demonstrate this “high-functionality” (Jarvis et al., 2017).

## Muscle recruitment

### Force magnitude

The muscle recruitment strategies with respect to maximum muscle force of the major hip muscles in able-bodied limbs and the intact limbs of individuals with UTF are broadly similar, with only a significant difference in pectineus force. However, there are significant differences in the plantar flexor muscle forces, Figure 3. The increased recruitment in the intact limb of individuals with transfemoral amputation during 50%–60% gait is in agreement with EMG studies (Mehryar et al., 2021; Wentink et al., 2013) which show increased recruitment of the plantar flexors in the intact limb of individuals with unilateral amputation to compensate for the for the amputated side.

It appears that muscle recruitment strategies in the residual limb group differs from the intact limb group and able-bodied limb group. Evidently, the psoas major and iliacus of the residual limb in individuals with UTF are heavily relied upon to actuate gait, with the first and third largest values of maximum muscle force across all muscles of all groups at 1.50 N/BW and 1.40 N/BW, these values appear to be abnormal (ie. Significantly larger than the able-bodied controls). This is indicative to the loss of the forward propulsion from the plantar flexors with the amputation, that has been reported as a reduced torque of the ankle on the prosthetic side (Seroussi et al., 1996). Therefore, the demand on these intact proximal flexors increases. It appears the adductor longus, tensor fasciae latae and pectineus may also be recruited to flex the hip in the residual limb for this reason. As shown in Figure 2, these muscles of the residual limb peak similarly to the iliacus and psoas major, at terminal stance phase/pre-swing phase of gait (40%–60%) when flexion action is required. These muscles have secondary flexor function (Neumann, 2010). This coincides with the increased peak in external extension moment of the residual limb group compared to the intact limb group, Figure 6. One study which recorded electromyography (EMG) of the residual limb during gait, found that more muscles were active and for longer periods during this “pre-swing” phase of gait in the residual limb compared to able-bodied controls (Wentink et al., 2013). The rectus femoris and sartorius, which are primary hip flexors in the intact anatomy/able-bodied anatomy, are compromised by the amputation surgery (Henson et al., 2021). This may increase the demand on the iliopsoas, adductor longus, tensor fasciae latae and pectineus. The force reached by these muscles in the residual limb group appears to be abnormal, as it is significantly higher than in the able-bodied group. This abnormal force requirement may have an effect on muscular endurance and therefore gait endurance (Fang et al., 2007).

The imbalanced recruitment of the iliopsoas between the bilateral limbs of individuals with UTF has not been reported in the literature. In fact, a study by (Harandi et al., 2020) estimated the opposite conditions with slightly higher levels of force in the iliacus on the intact side, yet comparisons are hard to make, because they included non-microprocessor knees and older participants in their study. As (Jarvis et al., 2021) highlights, this is a group representing a high functioning demographic compared to a more general UTF population. So, perhaps they exhibit a higher functionality which is demanding more of the iliopsoas. Due to the position of the iliopsoas, it is challenging to validate its activation with EMG data. Asymmetry in the lumbopelvic region has been associated with the increased levels of lower back pain in the transfemoral amputation population as an “mal-adaptive” impairment in this population’s gait (Devan et al., 2014), therefore the asymmetry found in the magnitude of iliopsoas contraction may have implications on secondary conditions such as lower back pain.

The elevated magnitudes of muscle force of the iliopsoas, adductor longus, tensor fasciae latae and pectineus in the residual limb may be unexpected due to the atrophy that is known to occur in the residual limbs post transfemoral amputation (Henson et al., 2021). However, when using normalisation methods which correct for thigh length, the maximum isometric extension, flexion, and abduction torque potential of the residual limbs of individuals with unilateral amputations is significantly greater than the intact, and equal to able-bodied control limbs (Sawers and Fatone, 2023). Furthermore, Sawers and Fatone (2023) found the hip adduction strength to be significantly greater in the residual compared to the intact and the control group, and suggested that this may be due to increased activation of the adductor muscles during gait. It may be that the increased recruitment of the adductor longus during gait found in our study contributes to the increased strength of hip adduction torque in this population.

Unlike the iliopsoas and other flexor muscles, the major extensor muscles in the residual limb appear to be recruited to a lesser magnitude than in both the controls and the intact side, Figure 1. The hamstrings are biarticular muscles in the intact state, yet they take a monoarticular function when cleaved and re-inserted in a transfemoral amputation (Henson et al., 2021). Due to this loss in function, they are found to be atrophied in the transfemoral residual limb (Jaegers et al., 1995). Although the gluteus maximus only articulates the hip, being the main hip extensor in the intact anatomy, it has also been found to be atrophied in the residual limb (Jaegers et al., 1995). The gluteus maximus inserts via the iliotibial tract which tends to require re-insertion in transfemoral amputation. So, although the individual’s residual limb anatomy will depend on the residual limb length and specifics of the traumatic injury and detailed surgical procedure used, the main hip extensors in the residual limb are all potentially functionally compromised by the surgery, which may explain their reduced recruitment compared to the intact and able-bodied limb groups. This imbalanced recruitment of the extensor/flexors on the residual limb side may impact pelvis stabilisation, and therefore pelvis kinematics. There is a peak in average anterior pelvis tilt at roughly 50% of the gait cycle for the residual limb group (Figure 6) which may be associated the recruitment of iliacus and the psoas major muscles (Figure 2) as their peak force and peak anterior pelvic tilt coincide.

The increased burden on the plantar flexors of the intact limb and of the hip flexors of the residual limb reinforces the theoretical need for powered prostheses, which generate energy to compensate for the power lost with the absent muscles in lower limb amputation. However, currently powered prostheses tend to be heavier than a microprocessor or mechanical prosthesis, increasing loading on the residual limb which limits their use, particularly for those with transfemoral amputation (Gehlhar et al., 2023).

### Impulse and peak activation

Together peak activation - force relative to maximum capacity - and impulse - an index of muscular energy expenditure - give an indication of the endurance implications of muscle recruitment (West et al., 1995; Bemben et al., 1996). The peak activation and impulse are abnormally high (ie. greater than the controls) for the flexor residual limb muscles highlighted previously: iliopsoas, adductor longus and tensor fasciae latae. This indicates a potential cause for reduced gait endurance. It has previously been shown that an exoskeleton on the residual limb providing external torque (flexion and extension) in the sagittal plane reduces the metabolic cost of gait by 15.6% for individuals with transfemoral amputations (Ishmael et al., 2021). The peak power injected in by the exoskeleton occurred at ∼60% of gait cycle assisting hip flexion with hip flexion torque (Ishmael et al., 2021). Considering the apparent increased energy expenditure of the hip flexors this may relate to the increased physiological cost of gait. Therefore, gait endurance could be improved if a similar functional ability was possible with reduced requirements on the flexor muscles (Ishmael et al., 2021). The estimations of peak activation tend to be higher in the residual limb which is indicative to the loss in muscular capacity (through atrophy and amputation).

Although the recruitment and function of the intact and able-bodied limbs appears to be similar, Figure 1 the impulse in the intact limb is significantly greater than the able-bodied for all main hip muscles apart from the adductor longus. This indicates that the intact limb is having to expend significantly greater energy to actuate gait compared to the able-bodied group and agrees with higher oxygen cost (Jarvis et al., 2017) and lower walking endurance (Linberg et al., 2013).

### Kinematic and kinetic interpretation

The participants with UTF amputation exhibited higher levels of anterior pelvic tilt compared to the controls and a significantly higher maximum external flexion torque and a lower maximum external extension torque in the intact limb compared to the residual limb, Figure 2. This is in agreement with a study of a similar population (Jarvis et al., 2021). This is consistent with the respective increased flexor muscle recruitment and lower extensor muscle recruitment in the residual limb. The frontal torque exhibits significant differences between the residual and the intact limb, with the external hip adduction torque on the residual limb being significantly smaller. This does not seem to be explained simply by differences in hip frontal kinematics Figure 6, further investigation into the relationship between differences in external loading (GRF) and differences limb kinematics is required.

### Limitations

There are several limitations to this study. Firstly, in terms of musculoskeletal modelling, the residual limb and prosthetic limb configuration was modelled as a rigid body composite “thigh” segment, neglecting pistoning and swivelling. The effect of this assumption is not known, yet a study with transtibial amputations found that including this relative motion had little effect on the magnitude of muscle forces predicted (Miller and Esposito, 2021). The anatomical geometries of the individuals with UTF were scaled from datasets of able-bodied participants and participants with bilateral transfemoral amputations for their intact and residual limbs respectively. Using anatomical datasets from the intact limb and residual limb of participants with UTF would have been more accurate. There may be some patterns in atrophy/hypertrophy that occur due to muscle recruitment patterns in gait in the UTF population that are not accounted for in this current model. Additionally, variations in surgical technique will impact the recruitment capabilities. There are two main methods of muscle stabilization, myodesis, where the muscles are sutured directly to the bone, and myoplasty, where the agonistic and antagonistic muscles are sutured together (Fabre et al., 2024). This variation was not considered in the anatomical dataset selection. Using anatomical geometries derived from participant specific MRI scans would have reduced error in muscle force estimations (Toderita et al., 2021b). Finally, the experimental data were obtained from two motion capture laboratories. Typically studies will present data from one laboratory only for reasons of consistency. However, in order to maximise participant recruitment and participation, a regional approach was taken by providing two laboratory locations. The laboratory technicians ensured a standardised calibration protocol was completed at each location and a consistent data collection protocol was followed.

## Conclusion

This study found that the UTF population have similar muscle recruitment strategies in their intact limb compared to AB controls. Muscle recruitment in the residual limb group varies significantly from the intact and able-bodied group, this is particularly true for the illiacus, the psoas major, the adductor longus and tensor fasciae latae. The demand on these muscles for flexor action appears to be elevated in the residual limb function due to the loss in forward propulsion from the loss of the plantar flexors. Although this group (fairly young, majority donning a microprocessor prosthetic knee, and majority ex-military) is likely to be of high functionality compared to a more general UTF population, there is room for improvement in their rehabilitation. To increase gait endurance and reduce the asymmetry in loading of the lumbo-pelvic region, which may be related to lower back pain, rehabilitation strategies to reduce this demand (on the residual and intact limbs) and to correct the asymmetry between muscle groups in the residual and intact limb of individuals with UTF should be developed.

## Data Availability

All data are available upon reasonable request, subject to the ethical approval and participant confidentiality. Requests to access these datasets should be directed to a.benton20@imperial.ac.uk.
